# Disrupted Dynamic Functional Connectivity of the Visual Network in Episodic Patients with Migraine without Aura

**DOI:** 10.1155/2022/9941832

**Published:** 2022-01-05

**Authors:** Heng-Le Wei, Tian Tian, Gang-Ping Zhou, Jin-Jin Wang, Xi Guo, Yu-Chen Chen, Yu-Sheng Yu, Xindao Yin, Junrong Li, Hong Zhang

**Affiliations:** ^1^Department of Radiology, The Affiliated Jiangning Hospital with Nanjing Medical University, Nanjing, Jiangsu 211100, China; ^2^Department of Neurology, The Affiliated Jiangning Hospital with Nanjing Medical University, Nanjing, Jiangsu 211100, China; ^3^Department of Radiology, Nanjing First Hospital, Nanjing Medical University, Jiangsu Province, Nanjing 210006, China

## Abstract

**Background:**

Visual symptoms are common in patients with migraine, even in interictal periods. The purpose was to assess the association between dynamic functional connectivity (dFC) of the visual cortex and clinical characteristics in migraine without aura (MwoA) patients.

**Methods:**

We enrolled fifty-five MwoA patients as well as fifty gender- and age-matched healthy controls. Regional visual cortex alterations were investigated using regional homogeneity (ReHo) and amplitude of low-frequency fluctuation (ALFF). Then, significant regions were selected as seeds for conducting dFC between the visual cortex and the whole brain.

**Results:**

Relative to healthy controls, MwoA patients exhibited decreased ReHo and ALFF values in the right lingual gyrus (LG) and increased ALFF values in the prefrontal cortex. The right LG showed abnormal dFC within the visual cortex and with other core brain networks. Additionally, ReHo values for the right LG were correlated with duration of disease and ALFF values of the right inferior frontal gyrus and middle frontal gyrus were correlated with headache frequency and anxiety scores, respectively. Moreover, the abnormal dFC of the right LG with bilateral cuneus was positively correlated with anxiety scores.

**Conclusions:**

The dFC abnormalities of the visual cortex may be involved in pain integration with multinetworks and associated with anxiety disorder in episodic MwoA patients.

## 1. Introduction

Migraine, which is a prevalent neurological disorder, is characterized by recurrent, unilateral, and moderate-to-severe headaches lasting from 4 to 72 hours and is often linked to clinical symptoms of photophobia, phonophobia, and nausea/vomiting [1]. The prevalence of migraine is reported to be approximately 12% in the world [2]. Each year about 2.5% of episodic migraine transform to chronic migraine [3], which is usually accompanied by more serious psychiatric comorbidities (anxiety, depression and cognitive disorder) and poorer outcomes and recovery, leading to decreased individual life quality and increased social burden [4, 5]. Photophobia is the most frequent presenting symptom in migraine with and without aura (MwA and MwoA) [6]. Moreover, migraine patients also feel discomfort of visual signal that which is well tolerant to healthy volunteers, even in the interictal period [7]. Then, the visual cortex may play a prominent and highly regulatory role in pain perception of migraine. Given the high prevalence of visual symptoms in patients with migraine, there is a substantial need for exploring the neurophysiological mechanism of the visual cortex to migraine, which may help develop novel methods to improve diagnosis and treatment.

Remarkably, resting-state functional magnetic resonance imaging (fMRI) has been instrumental in unlocking functional changes of the brain in neurological disorders, including migraine [8]. Recently, a growing number of resting-state fMRI studies revealed altered static functional connectivity (sFC) of the visual cortex in migraine. Tedeschi [9] and Wei [10] exhibited differences of neural activities in the visual cortex between patients with migraine and healthy participants and significant correlations between the visual-related sFC and psychiatric disorders. These results indicated the view that the visual cortex might not be only related to pain perception, but also associated with psychiatric disorders. Nevertheless, the aforementioned studies on the FC method are based on the assumption of the stationary pattern during the process of examination. Moreover, the theory has been demonstrated that the brain is a highly interconnected dynamic system [11]. Thus, the sFC pattern may distinctly limit to reflect the dynamic process of neural signal and ignore the potential changes, not easily detected, between the visual cortex and other brain areas. Resting-state dynamic FC (dFC) may be a useful neuroimaging method to overcome this static restriction and to explore time-varying patterns. More recent studies using the dFC method have proven high sensitivity in various neurological diseases, relative to the sFC analysis [12, 13]. Notably, a previous dFC study showed distinctly more neuroimaging information between migraine and persistent posttraumatic headache patients than sFC analysis [13]. Investigating time-varying FC will elucidate on the neural mechanisms underlying migraine, particularly from the aspect of temporal stability. Otherwise, migraine has also been shown to be associated with structural alterations in the brain. For migraine patients, MRI studies have revealed a distributed pattern of morphological brain abnormalities, which are characterized by increased and decreased gray matter volume regions [14]. Voxel-based morphometry (VBM) is a semiautomatic whole-brain method that has been shown to enable comparisons of gray and white matter between groups on voxel basis [15].

In the last decade, the resting-state fMRI analysis approaches, such as regional homogeneity (ReHo) and amplitude of low-frequency fluctuation (ALFF), have been used to depict the significant local neural characteristics of brain functions [16, 17]. Specially, ReHo reflects regional synchronization of spontaneous brain activities, and ALFF indicates regional intensity of spontaneous brain activities. Converging neuroimaging revealed that local and global properties of resting-state brain functions are not independent, but highly paired. This theory suggests that the regional dysfunction of ReHo and ALFF signals may implicit disruption of inter-regional FC. For this paper, we chose the MwoA patients, the most common subtype, to detect neuroimaging characteristics and to avoid patient heterogeneity. Moreover, the combination of ReHo, ALFF, and dFC measures may reveal neurophysiological mechanisms in MwoA comprehensively and may provide potential information synergistically than any measure alone. We hypothesized that abnormal variations of ReHo and ALFF values in the visual cortex are susceptive to the effects of recurrent headache attacks on MwoA patients. To our knowledge, this is the first study to perform dFC analysis to investigate the correlations between the visual cortex and clinical as well as psychiatric characteristics in MwoA patients.

## 2. Methods

### 2.1. Participants

Fifty-seven episodic MwoA patients at the neurology clinic at the Jiangning Hospital were enrolled. To control for the possible pharmacological and physiological effects, only MwoA patients who were drug-free for >1 month before the enrollment and headache-free for >3 days prior to and after the scanning were included in this study. Fifty age-, gender-, and education-matched healthy controls (HCs) were enrolled from community healthy volunteers. Diagnosis for each MwoA patient was established by two senior neurologists according to the 3^rd^ version of the International Classification of Headache Disorders (ICHD-3) [1]. General exclusion criteria for patients with migraine and HCs were (1) comorbid with nervous system disorders or other headaches, (2) any drug or alcohol abuse history, (3) women who were either pregnant or lactating, and (4) any MRI scanning contraindications. All participants were right-handed with at least nine years of formal education. The ethical committee of Jiangning Hospital permitted this study.

### 2.2. Clinical and Psychological Assessment

Episodic MwoA patients were subjected to various tests, such as the use of the Headache Impact Test (HIT-6) and Visual Analog Scale (VAS) scores to determine the impact intensity as well as severity of headaches and Physical Healthy Questionnaire (PHQ-9) and General Anxiety Disorder (GAD-7) scores to evaluate the symptoms of depression and anxiety, respectively. Every patient was required to keep a headache diary and completed a semistructured questionnaire on headache profile, demographics, and medical history during his or her first visit.

### 2.3. MRI Acquisition

A 3.0-T Philips MRI scanner (Ingenia, Netherlands) with an eight-channel head coil was used to MRI data acquisition. The patients were asked to reflex and close their eyes, but without falling asleep. Rs-fMRI images were obtained using echo-planar imaging sequence whose parameters were 36 slices, 2000 ms as the repetition time (TR), 30 ms echo time (TE), thickness of 3.5 mm, no gap, voxel size of 3.75 mm × 3.75 mm × 4.0 mm, 90° flip angle, 240 mm × 240 mm field of view (FOV), 64 × 64 data matrix, and 230 volumes. The fMRI sequence was completed in 8 min and 8 s. A 3D turbo fast echo T1WI sequence was used to obtain structural images. Its parameters were 170 slices, TR/TE = 8.1/3.7 ms, thickness of 1.0 mm, gap of 0 mm, FOV = 256 mm × 256 mm, 256 × 256 acquisition matrix, and FA = 8°. Structural sequences were completed in 5 min and 29 s.

### 2.4. Data Preprocessing

The Resting-State fMRI Data Analysis Toolkit plus V1.24 (RESTplus V1.24, http://restfmri.net/forum/) was used for image preprocessing, which included several steps: the first 10 volumes were discarded, slice timing correction with the 35th slice as the reference, head motion realignment, normalization of corrected images to Montreal Neurological Institute (MNI) space with a 3 × 3 × 3 mm^3^ resolution, regressing out the nuisance covariates (such as cerebrospinal fluid (CSF) signal, white matter (WM) signal, as well as 6 head motion parameters), linear detrending, 0.01–0.08 Hz band-pass filtering, and spatial smoothing using a 6 mm full-width half-maximum (FWHM) Gaussian kernel. Exclusion from analysis was done in case patients presented excess head motions in any direction > 2 mm or 2°. The mean framewise displacement (FD) was for each patient was determined to denote temporal derivatives of movement parameters.

Segmentation of cerebral tissues into the three tissue components (gray matter (GM), WM and CSF) was done by VBM analysis using the Statistical Parametric Mapping (SPM12) software (http://www.fil.ion.ucl.ac.uk/). An affine registration algorithm was used to register individual native-space GM segments to the standard Montreal Neurological Institute template. To refine intersubject registration, diffeomorphic anatomical registration through the exponentiated lie algebra (DARTEL) toolbox was applied to all participants' GM. Then, GM tissues were modulated via a nonlinear deformation strategy to enable comparisons of relative GM volumes adjusted for individual brain sizes. Lastly, smoothing of modulated GM volumes was done with a Gaussian kernel of 10 mm FWHM for whole brain statistical comparison. GM and WM volumes were determined by estimation of these segments. Brain parenchyma volume was the sum of WM and GM volumes.

### 2.5. ReHo and ALFF Calculation

Calculation of ReHo via Kendall's coefficient of concordance (KCC) was done using unsmoothed data. Local synchronization of time series of given voxels with those of its 26 nearest neighbors in a voxel-wise manner was determined. Then, division of individual ReHo maps was done based on their own global mean KCC for standardization. A Gaussian kernel of 6 mm FWHM was used to spatially smooth the standardized individual ReHo maps. Through fast Fourier transformation, unfiltered data were transformed into the frequency domain, and the power spectrum was obtained. The power spectrums' square root was averaged across 0.01–0.08 Hz at every voxel, with the obtained average square root recorded as ALFF. For standardization, ALFF of every voxel was divided by global mean of the ALFF value.

### 2.6. Seed-Based dFC Analysis

After ReHo as well as ALFF analyses, common impaired brain regions were selected as seeds and created using peak MNI coordinates with a radius of 5 mm. The Dynamic Brain Connectome Toolbox (V2.1 http://restfmri.net/forum/DynamicBC) was used for dFC analysis. A sliding-window strategy was used to created temporal dynamic patterns by convolving a rectangle with a Gaussian kernel (*σ* = 3TRs). According to prior research [18], the window size was 30 TRs (60 s), while window overlap was 90%, resulting in 64 windows per participant. The temporal correlation coefficient (*r*) between time courses for every seed and those of the rest of the brain voxels was determined in every sliding window. For every subject, several sliding-window correlation maps were obtained. For characterization of temporal variabilities in FC, coefficient of variation (CV) map over time of each voxel across each window was calculated to quantify the temporal variations, and the Fisher *Z*-transformation used to acquire variables similar to normal distribution.

### 2.7. Clustering Analysis

Assessment of reoccurring dFC patterns for all participants was done by the *k*-means clustering algorithm. We used squared Euclidean distance function to assess the similarity within all the sliding-window FC maps and estimated the counts of clusters automatically using the elbow criteria of cluster validity index. This index is the ratio between within- and between-cluster distance [19]. Then, the obtained clustering centroids were set as departure points for clustering all dFC windows from all participants.

### 2.8. Statistical Analysis

SPSS (version 25.0) was used for analyses, with *p* < 0.05 being the significance threshold. Demographic data and clinical variables were compared using two-sample *t*-tests for normally distributed continuous variables, while the Mann–Whitney test for nonnormally distributed continuous variables. The chi-square test was used for categorical variables between MwoA patients and HCs.

ReHo and ALFF comparisons were conducted by two-sample *t*-tests to establish between-group differences using a cerebral mask of the MNI template, gender, age as well as educational level controlled as covariates, and threshold set as *p* < 0.001, uncorrected. Subsequently, two-sample *t*-tests were performed to evaluate dFC differences between seeds and the rest whole brain in each state among the two groups, and significance was set at voxel level *p* < 0.001 with family wise error (FWE) correction together with a cluster extent threshold of 50 voxels. Partial correlations were performed, covaried for age, educational level, and gender, to estimate associations between the strength of brain functional impairment and clinical characteristics (*p* < 0.05) among MwoA patients.

To investigate the temporal characteristics of dFC patterns, dynamic characteristics, such as number of transitions (NT) and mean dwell time (MDT), were calculated. MDT was the mean time spent in a state prior to switching to another state. Moreover, NT represents the counts of transitions between different states.

## 3. Results

### 3.1. Demographic Variables

After the head motion check, two MwoA participants were excluded due to excess head motion artifacts. Differences in demographics such as age, education, and gender between HCs and MwoA patients were not significant. The mean FD also did not differ between the two groups (Table 1).

### 3.2. VBM, ReHo, and ALFF

For between-group comparison, the structural analysis did not reveal marked differences at corrected threshold for multiple comparisons (FWE correction, *p* < 0.05) or at uncorrected threshold (*p* < 0.001, cluster size > 100), with age, educational level, and gender as covariates without interest (Table 1).

**Figure 1:**
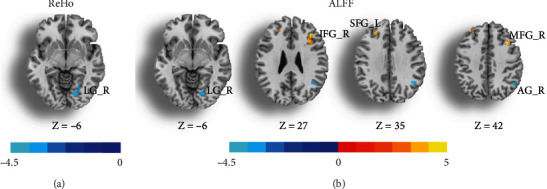
Statistic maps showing differences in the regional homogeneity (ReHo) (a) and amplitude of low-frequency fluctuations (ALFF) (b) values between patients with migraine without aura and healthy controls. The significance threshold was set at *p* < 0.001 (uncorrected) with a 20-voxel extension threshold. AG: angular gyrus; IFG: inferior frontal gyrus; LG: lingual gyrus; MFG: middle frontal gyrus; SFG: superior frontal gyrus; L: left; R: right.

Compared to the HCs, a two-sample *t*-test of ReHo maps revealed that MwoA patients exhibited low ReHo values at the right LG (Figure 1, Table 2). Moreover, ALFF values were markedly low in the right lingual gyrus (LG), angular gyrus (AG), and high in the left superior frontal gyrus (SFG), right inferior frontal gyrus (IFG), and right middle frontal gyrus (MFG) (Figure 1, Table 2).

### 3.3. Seed-Based Dynamic FC

Evaluation of ReHo as well as ALFF induced that MwoA patients had low values in the right LG than HCs. Then, right LG (*x* = 18, *y* = −75, *z* = −6, radius = 5 mm) was chosen as a seed to detect the dFC analysis with the whole brain.

**Figure 2:**
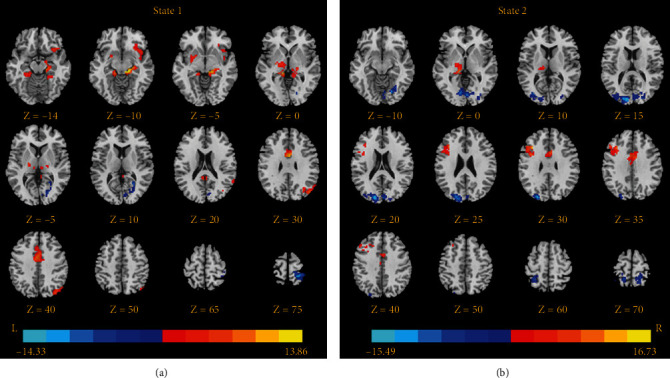
Brain regions showing dynamic functional connectivity differences based on the right lingual gyrus between patients with migraine without aura and healthy controls. The significance threshold was set at *p* < 0.001 with familywise error correction (cluster extent threshold > 50 voxels). The color bar, corresponding to the colors of connecting lines, indicates the *t* values of the two-sample *t*-tests between the two groups.

Table 3 and Figure 2 show the results from dFC analysis. For state one, MwoA patients exhibited lower dFC between the right LG and primary visual cortex (right calcarine sulcus) and primary somatosensory cortex (right postcentral gyrus (PoCG)), as well as higher dFC with the left thalamus, right insula, right parahippocampus/hippocampus (PHIP/HIP), right AG, bilateral middle cingulate cortex (MCC), and bilateral posterior cingulate cortex/precuneus (PCC/precuneus). For state two, MwoA patients also showed lower dFC of the right LG with some visual regions (bilateral cuneus and right fusiform gyrus) and primary somatosensory cortex (bilateral PoCG), as well as higher dFC with the left thalamus, left MFG and right MCC. Besides, between-group comparisons of NT and MDT in every state, based on right lingual gyrus seed, revealed that there were no marked differences.

### 3.4. Correlation Analyses

**Figure 3:**
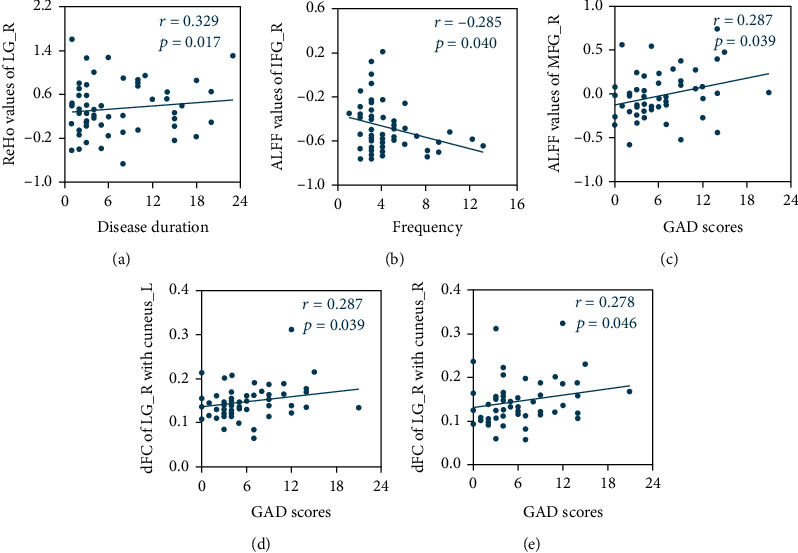
Correlations between abnormal functional features and migraine characteristics. A positive correlation between the ReHo values of right LG and disease duration (a) A negative correlation between the ALFF values of right IFG and headache frequency (b) A positive correlation between the ALFF values of tight MFG and GAD scores (c) Positive correlations between the dynamic functional connectivity of right LG with bilateral cuneus and GAD scores (d, e) The correlations were corrected for age, sex, and education. ALFF: amplitude of low-frequency fluctuations; dFC: dynamic functional connectivity; ReHo: regional homogeneity; IFG: inferior frontal gyrus; LG: lingual gyrus; MFG: middle frontal gyrus; L: left; R: right.

The ReHo values for right LG were associated with duration of disease (*r* = 0.329, *p* = 0.017). ALFF values for right IFG and MFG, respectively, correlated with headache frequency (*r* = −0.285, *p* = 0.040) as well as GAD scores (*r* = 0.287, *p* = 0.039). Furthermore, decreased dFC strengths between right LG and bilateral cuneus were positively correlated with GAD scores (left, *r* = 0.287, *p* = 0.039; right, *r* = 0.278, *p* = 0.046) (Figure 3).

## 4. Discussion

The current study explored differences in regional brain activity and internetwork dFC between MwoA patients and HCs using resting-state fMRI. The results disclosed that MwoA patients showed decreased ReHo and ALFF values in the right LG and disrupted dFC within the visual cortex and with other brain networks, such as default mode network (DMN), salient network (SN), central executive network (CEN), subcortical network, and sensorimotor network (SMN). Meanwhile, decreased dynamic functional alterations within the visual cortex were found significantly to be correlated with anxiety performance, which may indicate that the dysfunction of the visual cortex contributes to the emotional impairments in episodic MwoA patients during the attack-free period.

Patients with MwoA exhibited decreased ReHo and ALFF values in the right LG and decreased dFC within the visual cortex (right calcarine sulcus, bilateral cuneus, and right fusiform gyrus) in both two states compared to the HCs. The LG, calcarine sulcus, and cuneus are core primary visual cortex regions identified in previous resting-state study [20]. Accumulating evidence has indicated that the visual cortex is related to different high-order functions and suggested that the visual and visual-associated cortex might not only be involved in sensory integration and visual processing [21]. In addition, the current study revealed that ReHo values for the right LG were positively corrected with disease duration, consistent with previous results [10]. This study may suggest that the repeated headache attacks resulting in abnormalities of the LG belonging to the pain progressing and processing. However, studies on sFC within the visual network in migraine patients have reported conflicting conclusions. For instance, Tedeschi et al. [9] showed decreased activities of right LG in MwA patients compared to both MwoA patients and HCs during the interictal period. Likewise, many brain regions displayed higher sFC strengths in the right LG in MwoA patients than HCs through the independent component analysis method [22]. The controversial results manifest the heterogeneity among migraine subtypes and differences in postprocessing techniques may contribute to the previous discrepant findings. Moreover, the disruptions of dFC between the right LG and bilateral cuneus demonstrated significant positive correlations with anxiety scores in state two. Although the assessment of dFC of the visual cortex concerning psychiatric symptoms has lacked in interictal MwoA patients, previous static studies documented that the functional abnormalities of the LG and cuneus are specifically related to psychiatric disorders [23, 24]. Given the crucial role of the primary visual cortex above, these studies support our results, albeit partially, and reflect that intravisual microcircuit abnormalities play vital roles in the development and maintenance of headaches and show a meaningful correlation with psychiatric symptoms in MwoA patients during the interictal period.

For MwoA patients, dysfunction of the DMN has been previously demonstrated in resting-state fMRI studies [22, 25]. Nevertheless, current studies have not elucidated on dFC disruptions between the visual cortex and DMN. A large volume of literature has proven that the DMN is more active during task-free conditions and more inactive in goal-directed conditions [26]. Within DMN, the medial prefrontal cortex (MPFC) and AG and PCC/precuneus functions as the core regions are mainly responsible for fundamental roles in sensory information integration, pain chronicalization, attention switching, and cognitive demand [22, 27]. Buono et al. [22] showed hyperconnectivity within the DMN related to the LG and cingulate gyrus in MwoA patients, in good agreement with our own and no significant voxels in patients with migraine with aura relative to the HCs. Similar to our postprocessing methodological approach, Zhang et al. [25] exhibited significantly abnormal neural activity and correlation of ReHo values within the PCC with the intensity of migraine impact in interictal MwoA patients, whereas weaker sFC to the visual cortex. In line with these reports, we established that MwoA patients exhibited decreased ALFF values in the right AG, whereas increased dFC between the visual cortex and DMN (right AG and bilateral PCC/precuneus) in state one. Moreover, there were significant correlations between years with headache and sFC and between pain intensity and dFC of DMN in patients with migraine [13]. The present study elicited that altered dFC between the visual cortex and DMN is a distinctive characteristic in interictal MwoA patients, suggesting the differences of static and dynamic connectivity patterns may explain the discrepant findings.

In addition, MwoA patients exhibited increased dFC between the right LG and ipsilateral insula in state one compared to the HCs. The insula is often referred to as a core region of SN, a pivotal large-scale network that is associated with task processing, attention processing, and integrating of external and internal stimuli [28, 29], as well as gating pain processing [30, 31]. In accordance with the present results, some clinical and animal model studies have concluded that the insula is involved in regulation of headache intensity and attack frequency of migraine and other neuropathic pain [32–34]. However, a prior study [35] demonstrated contrary results in female MwoA patients during a headache-free period. Besides, the present outcome is contrary to that of Niddam [36] who found the decreased static connectivity between the key node of the SN and visual cortex correlated with the headache severity in MwA patients between attacks but not in MwoA patients. These differences may be the results of sexual diversity [37] and time-varying courses. On the basis of the above information, the dFC may discover more features of neural spontaneous activity and visual-SN dFC pattern may suggest the specificity to episodic MwoA patients in the interictal period.

Besides, the SN has been suggested as a salient detector to monitor the stimuli for further processing in other relevant large-scale networks [29] and then enables a switch between triggering the activation of task-positive network and disengaging from the task-negative network in case a salient event is noticed [38]. Researchers have put forth a “triple network model” based on analysis of large-scale networks, including the DMN, SN, and CEN and participate in almost sensory, affective, and cognitive processing [39]. Interestingly, the significant regions detected in the current study with greater neural spontaneous activity and with increased dynamic time course among the visual cortex were located within the dorsolateral prefrontal cortex (DLPFC), a core region of the CEN, in MwoA patients. Moreover, the study displayed significant correlations between the increased neural activity within the right DLPFC and attack frequency as well as anxiety scores, suggesting that DLPFC-related multinetwork circuits could participate in the processing of pain and be linked to anxiety symptom, underlying the pathophysiology of neuropathic pain [40] and psychiatric disorders [41].

Our findings have demonstrated that visual-related modulation of pain is associated with multidimensional networks. These networks, thalamus, and brainstem constitute the hyperexcited trigeminovascular pathway underlying the pathogenesis of the migraine attack [42]. The peripheral trigeminal sensory afferents converge onto the trigeminal cervical complex and then project to the brainstem, thalamic, and cortical regions to process nociceptive inputs from the trigeminovascular system. The brainstem nuclei involved in initiating the painful perception of the trigeminal sensory pathway are the PAG and rostral ventral medulla (RVM) [43]. The PAG-RVM circuit has pivotal roles in pain trigger of facilitation and inhibition, significant functional coupling\interconnection with the limbic system and thalamus, and modulation of attack frequency in patients with migraine [44, 45]. When contrasting MwoA patients versus HCs in this study, patients with migraine had higher dFC with subcortical regions (i.e., hippocampus, cingulate cortex, and thalamus), whereas decreased dFC was seen with the primary sensory cortex. The thalamus, as the biggest relay region, receives efferent connections from the brainstem, especially from the PAG, and projects signals to the higher-order cortex via specifically anatomical and functional features [46, 47]. In accordance with the present results, our previous study also showed increased sFC between the visual cortex and thalamus and somatosensory cortex correlated with psychiatric comorbidity and migraine characteristics [10, 48] in interictal MwoA patients.

Moreover, the limbic system, including the amygdala, angulate cortex, hippocampus, hypothalamus, and partly PFC, overlaps with the Papez circuit hypothesized to underlie the expression of psychiatric disorders [49]. In this regard, the processing of emotion and descending pain pathways may share the common neurophysiological circuits in patients with migraine. Negative emotional stimuli induced increased activation in the limbic system, thalamus, PFC, and visual cortex [50]. Further, an arterial spin labeling study directly reported neural correlations of pain intensity mapped to limbic-affective circuits and heightened the importance of psychiatric disorders in chronic pain [51]. Thus, the migraine neural model must be responsible for nociception as well as stress, emotion, and psychiatric comorbidity. To this end, beyond the neurovascular model, a neurolimbic pain network model has been proposed to explain the role of limbic dysfunction and cortical hypersensitization [52]. Taking these pieces of evidence into consideration, we can infer that the limbic system participates in the perception of nociceptive inputs and modulation of psychiatric comorbidity in patients with migraine through the trigeminovascular pathway, perhaps accounting for the dynamic bidirectional influence of nociception and psychiatric disorders.

Some important limiting factors of our study have to be considered. First, we only analyzed changes in FC and ignored the deviations in structural connectivity of brain regions as well as causal relationships between functional and structural alterations. We expect further studies to increase the sample size, detect structurally vulnerable changes, and confirm our results. Moreover, the only parameter discussed is the dFC variance, and more parameters should be focused on to validate the potential of altered dynamic alterations as clinical biomarkers in interictal MwoA patients. Furthermore, the causality relationship between psychiatric disorders and dFC could not be explored in this cross-sectional study. To definitively answer this question, future longitudinal studies should be designed to verify the causal mechanisms between emotion and resting-state FC. Finally, another potential confounding factor is that the patients were enrolled with medication-free for at least one month, but the potential physiological effects of taking medication over the long-term on the brain signal are unknown in this study.

## 5. Conclusion

In conclusion, we demonstrated the dynamic functional interaction of the visual cortex within itself and to other core large-scale networks in interictal MwoA patients. These changes may suggest the visual cortex plays a crucial role in the neurophysiological features and account for modulation of nociceptive inputs in MwoA patients. Moreover, the results shed light on the mechanisms of neural circuits of aberrant dynamic functional networks correlated with psychiatric disorders. We hope that the findings might provide a novel perspective for the development of individualized therapeutic targets of the neural pathway in migraine patients.

## Figures and Tables

**Table 1:** Demographic and clinical characteristics of all participants.

	MwoA	HCs	*p* value
Age (years)	33.58 ± 10.88	37.26 ± 10.28	0.079
Sex (male/female)	7/48	6/44	0.910
Education (years)	12.56 ± 3.10	12.20 ± 3.55	0.576
Disease duration (years)	7.22 ± 6.01	/	/
Frequency (days/month)	4.35 ± 2.53	/	/
HIT-6 score	59.85 ± 8.96	/	/
VAS score	6.49 ± 1.35	/	/
GAD-7 score	6.22 ± 4.58	/	/
PHQ-9 score	7.38 ± 5.24	/	/
Framewise displacement	0.22 ± 0.08	0.19 ± 0.08	0.190
Gray matter (mm^3^)	628.31 ± 55.31	605.77 ± 55.02	0.139
White matter (mm^3^)	487.13 ± 43.93	496.04 ± 53.40	0.351
Brain parenchyma (mm^3^)	1115.44 ± 89.60	1101.81 ± 92.82	0.446

Data are presented as mean ± standard deviation. GAD-7: General Anxiety Disorder; HIT-6: Headache Impact Test; PHQ-9: Patient Health Questionnaire; VAS: Visual Analogue Scale; MwoA: migraine without aura; HCs: healthy controls.

**Table 2:** Significant brain regions in ReHo and ALFF values between MwoA and HCs.

Regions	MNI coordinate			Cluster size	*T* value
	*X*	*Y*	*Z*		
*ReHo*					
Lingual gyrus_R	18	-75	-6	22	-4.114
*ALFF*					
Lingual gyrus_R	18	-75	-6	27	-4.619
Angular gyrus_R	48	-57	39	44	-5.347
Inferior frontal gyrus_R	36	30	27	31	5.853
Superior frontal gyrus_L	-24	33	33	24	5.426
Middle frontal gyrus_R	36	15	42	25	5.694

Significance thresholds were set at a voxel-level *p* < 0.001 (uncorrected). ReHo: regional homogeneity; ALFF: amplitude of low-frequency fluctuation; MwoA: migraine without aura; HCs: healthy controls; L: left; R: right.

**Table 3:** Regions with differences in dFC of right lingual gyrus between the MwoA and HCs.

Regions	MNI coordinate			Cluster size	*T* value
	*X*	*Y*	*Z*		
*State 1*					
Calcarine sulcus_R	27	-57	9	110	-12.707	
Angular gyrus_R	45	-75	30	192	13.030
PCC/precuneus_B	-3	-45	21	67	10.858
Insula_R	36	18	-12	92	11.359
Thalamus_L	-12	-33	0	158	14.499
PHIP/HIP_R	12	-30	-9	177	16.732
Middle cingulate cortex_B	0	3	30	221	14.958
Postcentral gyrus_R	21	-39	78	125	-15.489
*State 2*					
Cuneus_L	-12	-90	15	304	-14.333
Cuneus_R	3	-87	21	102	-11.280
Fusiform gyrus_R	21	-72	-9	169	-12.643
Middle frontal gyrus_L	-33	21	33	226	13.863
Thalamus_L	-15	-30	3	85	12.343
Middle cingulate cortex_R	6	0	33	95	11.228
Postcentral gyrus_L	-18	-48	69	98	-11.208
Postcentral gyrus_R	18	-51	72	65	-10.420

Significant threshold was set *p* < 0.001 with family wise error (FWE) correction (cluster size > 50 voxels). dFC: dynamic functional connectivity; PCC: posterior cingulate cortex; PHIP/HIP: parahippocampus/hippocampus; MwoA: migraine without aura; HCs: healthy controls; B: bilateral; L: left; R: right.

## Data Availability

The datasets used and analyzed during the current study are available from the corresponding author on reasonable request.
